# Urban street names: An opportunity to examine biocultural relationships?

**DOI:** 10.1371/journal.pone.0200891

**Published:** 2018-07-25

**Authors:** Charlie M. Shackleton

**Affiliations:** Department of Environmental Science, Rhodes University, Grahamstown, South Africa; Auburn University, UNITED STATES

## Abstract

With increasing urbanisation there is concern regarding loss of experience and knowledge of biodiversity amongst urban populations. Yet biodiversity representations are retained in many art and functional forms, including names of places, buildings, institutions and streets. These manifestations offer a window to examine the relationship between humans and their experienced or imagined environment using a biocultural lens. I quantified the current prevalence of urban streets named after animals or plant species, the diversity of species represented, whether they are native or non-native, whether representative of the biome in which the town was situated and the change in prevalence through time. The street names of 48 towns in a one degree wide south-north belt across seven of South African’s biomes were captured and analysed. Of the 4,359 street names, 11.1% were named after plants (218 species) and 5.3% after animals (131 species), although some towns had none and others more than 40%. Approximately half of the plants were native to South Africa, whereas over 80% of the animals were. There was no correspondence between the species composition reflected in street names and the biome in which towns were located. The proportion of streets named after plants or animals has generally increased over the last two hundred years. These results provide insights into the bioculturally defined plants and animals that are valued by past and present urban communities, showing that they are generally from a wider array than can be found or experienced in the local setting.

## Introduction

Street names fulfil a number of functions, the most obvious one being to assist in navigation or identification of a specific location. Additionally, street names are commonly also a means of commemorating specific events or individuals, which help promote historical figures or noteworthy events in public consciousness on a daily basis. However, who or what is commemorated is a social and frequently political decision [1–3]. Consequently, the names of some streets are contested [4], and may change when one political order or dominance is replaced by another [5], sometimes resulting in the use of more than one name for the same place or street, the old and the new [3,6]. Street names may also be used to differentiate communities or sections of towns and cities for social, political or financial purposes [1,3]. However, the meaning of many street names diminishes with time, with current generations no longer aware of the origin of the name or any alternative interpretations or values associated with it [1]. In this way, street names also have an educational purpose if their origin and meaning are kept alive.

Although most streets are named after people, events or places [1], local or national heritage is not confined solely to historical events or well-known persons. Heritage also encompasses aspects of the natural world which have meaning to local populations and may contribute to a sense of place and identity [7,8]. Natural features and places with meaning to past or present populations become named and the names are absorbed into local narratives, identity and meaning [9]. In the same manner, streets in urban settings may assume names after particular current or former natural features in the local vicinity (e.g. River street, Park road, or Greenhill drive) or species of significance in local culture or folklore, or were historically abundant in that area (such Elephant street or Protea drive), adding to local sense of place [6].

A particular dimension of potential relevance to biocultural diversity is the representation of plant and animal names (hereafter referred to as biodiversity street names) in street names. Naming a street after a particular animal or plant communicates those elements of biodiversity that local people hold (or held) in some regard, one indicator of cultural keystone species [10]. In former colonial towns biodiversity street names in the older sections may possibly disproportionately reflect species from the colonial home country, expressing the settlers’ desires for and memories of their formative years in another country. These species have little meaning to modern day urban populations, many of whom may never have seen the non-native plant or animal in question, yet are encumbered with the name, an anachronistic legacy of colonial power. In this way, seemingly neutral biodiversity street names may also be contested. Why one species rather than another? Who gets to select which species? In what language are the species names presented? Some species have cultural significance to certain societies or groups, and hence may be favoured over proposals for alternative fauna or flora, and the environment is often a site for multiple world views and at times competing cultural notions [11–13]. Yet homogenising processes of globalisation and biological invasions may further impinge on local identities expressed in place names [9], including streets. For example, there is an Elephant Street in London (UK), a Camel Street in Clayton (North Carolina, USA) and a Lantana Street in both Cape Town (South Africa) and Ivanhoe (Australia), countries which were never home to these species. On the other hand, there are inextricable links between natural and cultural diversity [12,14,15], which can result in a greater richness of biodiversity names in biologically diverse regions [9] as expressions of past (and perhaps current) associations and processes between humans and natural features which have shaped both [16]; whether this pertains to street names remains to be explored. Botzat et al.’s [17] review indicates that indeed, such exploration is required across a range of urban settings as the cultural dimensions and variations in biodiversity interpretation, appreciation and use have rarely be analysed in urban environments.

This mutually reinforcing shaping of views and uses of the natural and cultural domains is encapsulated in the concept of biocultural diversity. First coined to describe and provide analytical frameworks to explore the nature-culture co-dependencies of native peoples in insular locations [18], it is increasingly invoked across all settings, including modern urban societies [19]. The application to a broader suite of settings rests on the conceptualisation of culture, and consequently heritage, as temporally and spatially dynamic [12]. Culture provides the filter or the lens through which individuals, communities and societies view and interact with biodiversity. Culture facilitates what activities are possible and what viewpoints are accepted within different social groups (at a particular period and location), and thus circumscribes how the natural environment is experienced and lived [20]. Importantly this facilitation changes as external contexts and actors change, presenting culture as a selective force [12] which may transform rapidly or slowly depending on the context and the nature and magnitude of the transforming drivers. Some cultural ideas and practices may be readily transposed from one setting to another, whilst others may persist only in certain settings. With such a dynamic context, it is inevitable that biocultural diversity is equally dynamic in form and process [19]. Street names therefore offer an intriguing window into biocultural expressions of how current and previous urban communities (increasingly multicultural and increasingly divorced from direct experiences with biodiversity), view and value the natural world and how that has mutated through time.

Besides the cultural and heritage roles of biodiversity street names, they also offer an educational function, which is particularly pertinent in an increasingly urbanised world wherein many urban residents have limited and declining contact with biodiversity [20–23]. What proportion of residents can actually identify the flower, tree or animal reflected in the name of the street on which they live or frequently travel along? The proportion is likely to be particularly low if the flower, tree or animal is not native to the country or region, culturally or economically meaningful or globally charismatic [24]. Therefore, naming streets after biological species potentially helps to contribute in creating awareness of or remind urban residents about biodiversity, but this needs to be explored. It is possible that the occurrence of biodiversity street names will be more common in larger towns and cities as they have more streets which allows naming to move beyond mostly significant persons or events.

Within the context of the above, the aim of this work was to determine the prevalence and nature of streets named after biodiversity in South African towns. More specifically I sought to answer the following five questions: (1) What is the prevalence of street names reflecting biodiversity? (2) What is the diversity of such street names? (3) Are the biodiversity street names reflective of the biome in which the town is situated? (4) Has the prevalence of biodiversity street names changed through time? and (5) To what extent are South Africa’s national floral and faunal species represented in street names? Related to these I considered the following three hypotheses. First, given that animals are usually more charismatic to the general population than plants [25], and that many African households have totemic animals [26], that there would be a greater prevalence of animal street names than plant ones. Second, with increasing environmental awareness and concern internationally [27], the prevalence of biodiversity street names would be higher now than in the past. Third, through promotion of national heritage and pride the national heritage biodiversity symbols (national flower, tree, animal, etc.) would be amongst the most prevalence species reflected in biodiversity street names.

## Methods

In examining the prevalence and nature of biodiversity street names it is useful to first understand how street names are currently given in South Africa. Indeed, as a country that has only recently emerged from a long racially defined colonial and apartheid past, there is much interest in and at times contestation of, street names in South Africa [6,28]. Currently, naming of streets is the responsibility of the municipal council in which a town or city is located. Councillors are elected by residents every five years. Proposals for names for new streets are usually suggested by the development company planning a specific site, by members of the public or by council members. Irrespective of who proposes a specific name, it needs to be debated and approved by the town council. The same applies with applications for renaming an existing street with the difference that any proposed changes must be first advertised and open for public comment, and the recommendation of the town council must be endorsed by the South African Geographical Names Council.

**Fig 1:**
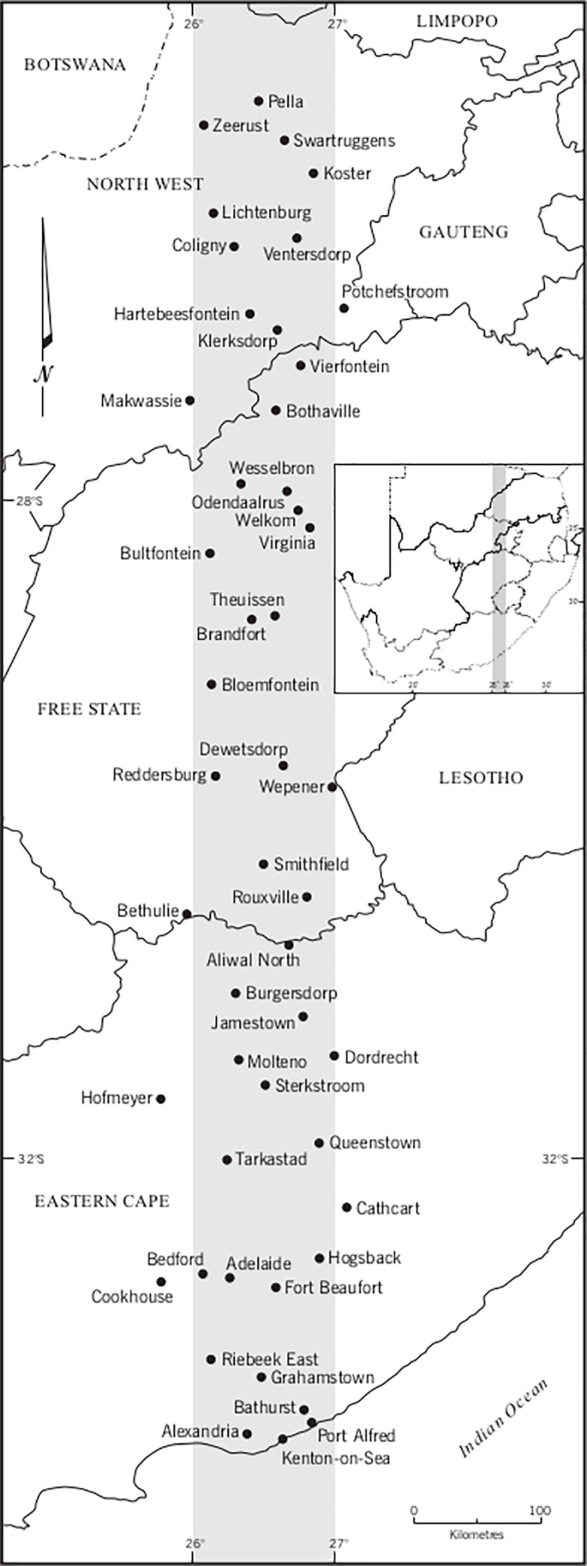
Location of the 48 sample towns in the south-north belt between 26^0^ and 27^0^ east in South Africa.

All towns and cities (hereafter referred to as towns) occurring in a 1 degree wide belt (between 26^0^ and 27^0^ East) from the southern coast of South Africa on the Indian Ocean to the border with Botswana in the north (a linear distance of approximately 1,100 km) were sampled (Fig 1). The base map for identifying sample towns was the 1: 1 000 000 vegetation map of South Africa [29]. The transect included six of the seven vegetation biomes in South Africa (forest, fynbos, grassland, nama karoo, savanna, thicket) and three of its nine provinces. The street map of each town was accessed via Google Maps during March and April 2016 and the names of all, or a sample of, streets were manually recorded in an Excel spreadsheet.

A few of the small and remote towns did not have street names recorded in Google Maps and so were excluded (except Hogsback for which we obtained a local tourism map). Similarly, a very small number of streets (< 1%) did not have a name in Google Maps and so were not part of the analysis. Four large towns (populations 150,000–500 000) were situated in the transect, each with thousands of street names, which would disproportionately dominate the final analysis. Therefore, in each of these towns a sample of streets was taken, following the south-north transect approach adopted for selection of towns. For each of these four large towns, a 1 km wide transect was sampled along the longest south-north axis of the town. The names of all streets crossing or within the 1 km wide transect were recorded. By the end of data collection the final sample was forty-eight towns and 4 359 street names, ranging from nine names in the smallest (Hofmeyer and Vierfontein) to 426 names in Queenstown. The reported means and standard deviations were calculated across the sample of 48 towns unless other stated. The population size across the 48 towns ranged from 780 to approximately 0.5 million. Population sizes for each town were taken from the city population website (http://www.citypopulation.de). Whilst there are likely to be some inaccuracies, the level should be consistent across towns.

The name of each street in the database was then classified according to whether it represented a biophysical, biodiversity or environmental theme or not. The categories were plants, animals, aquatic species, topographic features, mineral, astronomical or none of these. In some instances a particular name could be interpreted as falling into both a biophysical category as well the non-biophysical category. For example, a street named Rose might be the surname of a prominent or founding citizen of the town, it might be the first name of a female or it might be the name of a flower. Similarly, Orange might be the name of a fruit, a colour or the name of the largest west-flowing river in South Africa. In such instances I adopted an inclusive interpretation, i.e. assigned it to one of the biophysical groups rather than the non-biophysical one, unless the very local context indicated otherwise. By this I mean that at times the names of neighbouring streets offered guidance. For example, if the street named Rose was flanked by streets with other typically female first names (such as Mary or Henrietta), then I classified Rose as non-biophysical. But if flanking streets had other flower names then the assignment to the botanical category was supported. Twenty-four names (0.6%) were composites spanning more than one category. These were assigned to the category reflecting the last word of the composite name. For example, Kameeldoringfontein (Camel Thorn Spring) would be assigned to the aquatic category rather than botanical one as represented by the camel thorn tree (*Acacia erioloba*) or Grassy Hill would be assigned to the topographic category rather than botanical. In four instances I encountered different street names for different sexes of the same species (lion/lioness) or juvenile and adult stages (cow/calf; goat/kid; lion/lion cub). In all these instances the sex or age differences were ignored when counting the frequency of occurrence. All results are presented as means ± standard deviation unless otherwise stated.

As a multilingual country (South Africa has eleven official languages) street names were in one of several different languages reflecting the most common languages spoken in specific regions of the country. English and Afrikaans street names were evident in every town, reflecting the country’s colonial past. Additionally, in the Eastern Cape province isiXhosa names were common, in the Free State province SeSotho names were widespread and in the Northwest Province, Tswana names were frequently encountered. Consequently, classification of street names in different languages was undertaken by two vernacular language speakers for each language. After the classification had been done, the few differences between the two people who did the categorisation were identified and discussed as to what the final classification should be. The determination of the frequency of any specific name took translations into account. For example, the final count of five for the street name Elephant was derived from Elephant (1), Olifant (Afrikaans) (1), Ndlovu (isiXhosa) (2) and Tlou (Sepedi and Tswana) (1).

Five of the larger towns within the Eastern Cape province that have a library or museum were selected to examine if there had been any change in the prevalence of biodiversity street names through time. The Eastern Cape was chosen because of the three provinces covered in the cross-country belt transect, it has the oldest history of colonial settlement and mapping. Old maps of each town were accessed and street names recorded. The maps were grouped into approximately 50 year time periods. The prevalence of biodiversity street names was then determined in the same manner as the rest of the study. This approach was not without drawbacks. Firstly, not all towns had maps for each 50 year period and so the sample size varied per half-century intervals. Secondly, not all historical maps for a specific town included all sections of the town.

Data were captured in Ms Excel and imported in Statistica v13.3 for analysis. Regression analysis was used to assess any relationship between the number of animal and plant street names per town, as well as between town population size and the incidence of animal and plant street names. To address the third research question, the representivity of the street names of the biome in which each town was situated was assessed using Canoco for Windows 5. Data were first log-transformed to eliminate the influence of extreme values on ordination scores. Principal component analysis (PCA) was performed to investigate how biomes influenced species composition, using presence/absence data. Each town was treated as a sample and the species occurring as street names were the species composition of the sample. Each town was coded for the biome in which it occurred.

## Results

**Fig 2:**
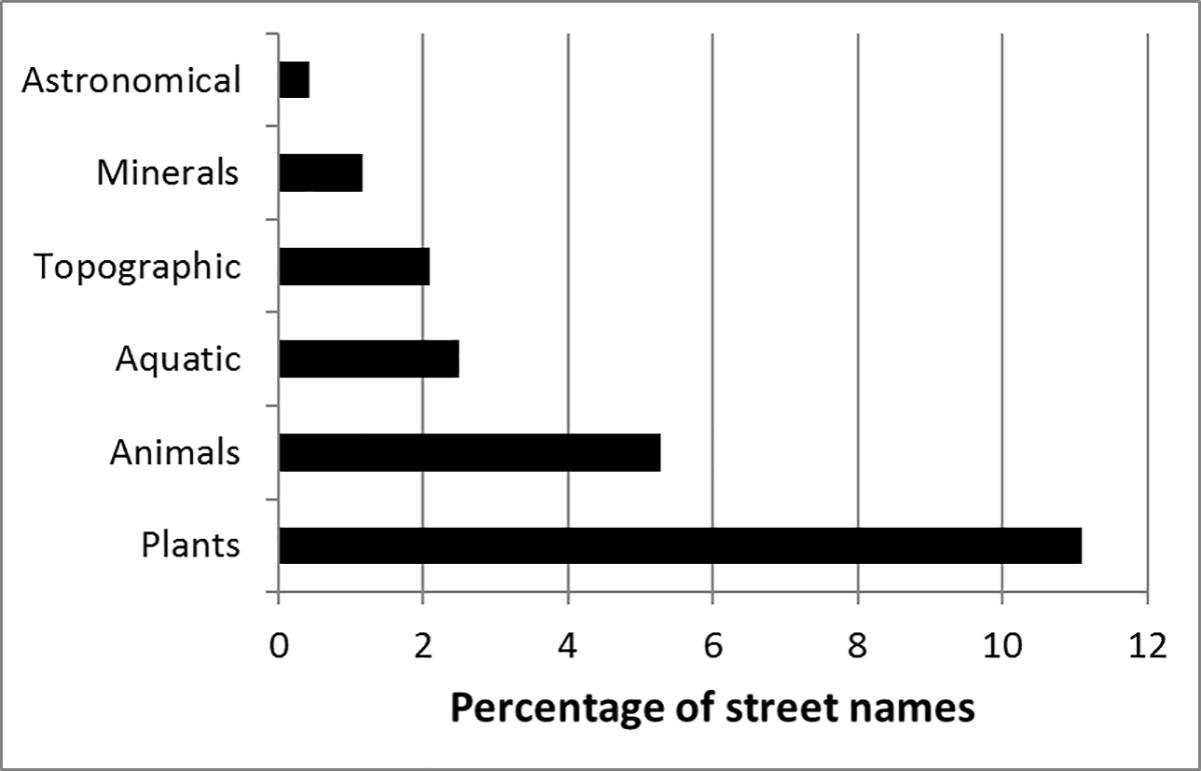
The proportion of 4,359 street names in 48 South African towns that reflect a natural environment theme.

Slightly less than one-quarter (22.6%) of the 4,359 street names represented species or features of the natural world (Fig 2). Within this group, the most common were streets named after plants (11.1%) or animals (5.3%). A similar proportion (22.9%) of the 48 towns themselves were named after biodiversity features, but the most common (63.6%) of these related to aquatic features such as springs or streams (e.g. Potchefstroom, Bloemfontein).

Considering streets with biodiversity names, a greater number had plant names (484 streets) than animal names (230 streets). At the town scale plant names represented on average 10.4 ± 10.6% (mean ± std dev.) of street names, ranging from 0 to 42.6%. Corresponding figures for animal street names were 2.7 ± 4.1%, ranging between zero and 17.2%. There was no relationship indicated from the regression of the proportion of plant and animal street names within towns (r = 0.084; F = 0.324; df = 1, 46). Nor was there any relationship between town population size and the proportion of streets with plant (r = 0.103; F = 0.501; df = 1, 46)) or animal (r = 0.068; F = 0.211; df = 1, 46) names. The number of streets named after native plants and non-native plants was similar, as was the case with the number of native and non-native species encountered (Table 1). In contrast, 80% or more of animal names and streets with animal names were of native species.

**Table 1:** The percentage of streets with biological names reflecting native, non-native and non-species level names in 48 towns in South Africa.

Group	Streets or species	No. of streets	Count	%
Plant names	No. of streets	Native plants	214	44.2
		Non-native plants	222	45.9
		Non-species level plant features (e.g. park, wood, bush)	48	9.9
		Total	484	100.0
	No. of species	Native plants	91	41.7
		Non-native plants	96	44.0
		Non-species level plant features	31	14.2
		Total	218	100.0
Animal names	No. of streets	Native animals	195	84.8
		Non-native animals	30	13.0
		Non-species level animal features (e.g. den, bird, horn)	5	2.2
		Total	230	100.0
	No. of species	Native animals	106	80.9
		Non-native animals	22	16.8
		Non-species level animal features	3	2.3
		Total	131	100.0

The most common plant names were oak, carnation and protea (Table 2). Correspondingly, the most common animal names were lion, duiker and eland (Table 2). Of the 14 most common plant names, six were of native species. Collectively, the 14 most common plant species accounted for 125 streets, of which 41.2% were native. Similar data for animal species reflected that 12 of the most common 14 species were native, representing 87.6% of the streets bearing the name of any of those animal species (69 streets in total). Across the entire database, most of the animal names were mammals (51.3%) or birds (33.3%). A few fish were named, but only one was a freshwater species (catfish). Only one street was named after an insect (Fly Street) and two after reptiles (tortoise, iguana). None were named after arachnids. The list of plant names was more diverse, covering most life forms such as trees, ferns, shrubs, perennial and annual flowers and succulents. Several fruit or nut trees were represented in street names, as well as a few herbs and spices, but no street was named after a vegetable.

**Table 2:** The 14 most common plant and animal names encountered as street names in 48 towns in South Africa (standardised to English names) (n = native to South Africa; a = non-native species).

Taxon	Species	Status	Count
Plants	Oak	a	12
	Protea	n	11
	Carnation	a	10
	Olive	n	9
	Blue gum	a	9
	Dahlia	a	9
	Jacaranda	a	9
	Mimosa	a	9
	Aloe	n	8
	Karee	n	8
	Willow	n	8
	Yellowwood	n	8
	Pine	a	8
	Rose	a	7
Animal	Lion	n	12
	Duiker	n	6
	Eland	n	6
	Cow	a	5
	Crocodile	n	5
	Elephant	n	5
	Falcon	n	5
	Springbuck	n	5
	Dove	n	4
	Gemsbuck	n	4
	Kudu	n	4
	Peacock	a	4
	Steenbuck	n	4
	Swallow	n	4

South Africa has a suite of species as national symbols, but they were not well represented within the street names encountered. The national animal is the springbuck (*Antidorcas marsupialis* Zimmermann) which, with five occurrences, was tied fourth on the list. The national flower is the king protea (*Protea cynaroides* (L.) L.), which was not recorded for any street. Protea was a common street name, but could reflect any of the approximately 90 protea species in the country. The national tree is the real yellowwood (*Podocarpus latifolius* (Thunb.) R.Br. ex Mirb.), which like the protea, was not specifically mentioned, although yellowwood (four species in the country) was not uncommon as a street name. The national bird (blue crane; *Anthropoides paradiseus* Lichtenstein) and fish (galjoen; *Dichistius capensis* G.Cuvier) were poorly represented with two (crane) and zero streets, respectively.

**Fig 3:**
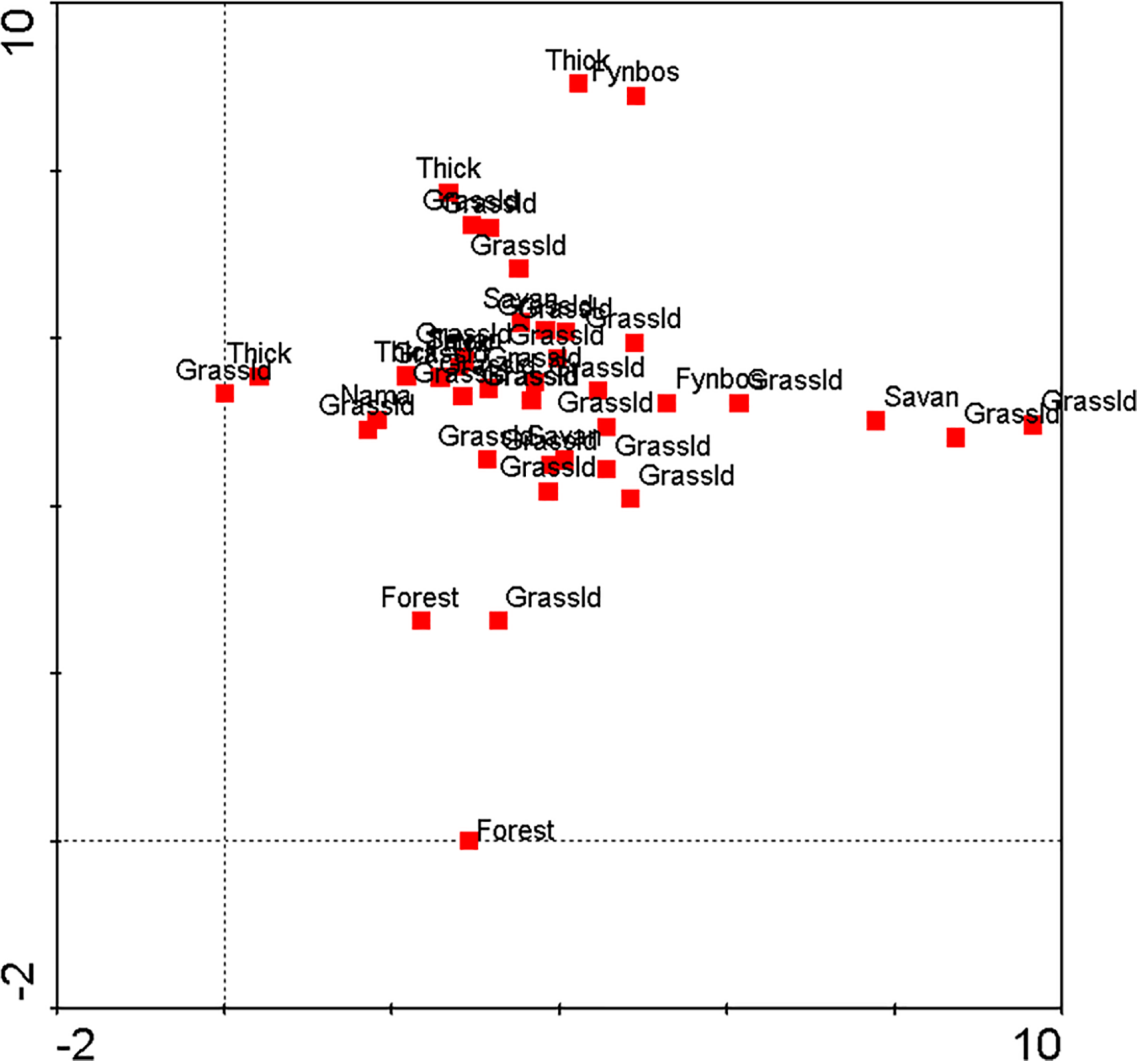
Principle components analysis scatter plot of the biome of the 48 sample towns based on the species composition of the street names.

The PCA revealed no distinct grouping of towns within biomes (Fig 3). Rather, the different biomes were interspersed among one another. This indicates that the composition of street names were generally not a reflection of the biome in which the town was situated.

The time trend data showed that the proportion of streets named after plants has increased, in towns in the Eastern Cape province, especially after the mid-20^th^ century (Table 3). The trend in animal named streets was variable, peaking in the latter half of the 20^th^ century, and is currently below that peak.

**Table 3:** Prevalence of plant and animal street names in five Eastern Cape province towns for five periods from pre-1850 to post-2000 (dashes mean no map was available for that period; zeros mean maps were available but none of the street names for that period were of animal or plant species).

Town	Taxa	Period				
		<1850	1850–1900	1901–1950	1951–2000	>2000
Aliwal North	Plant	0	-	1.4	-	4.5
	Animal	0	-	1.4	-	3.2
Fort Beaufort	Plant	0	0	0	-	26.6
	Animal	0	0	0	-	0
Grahamstown	Plant	0	0	1.1	2.7	5.2
	Animal	3.6	0	0	3.8	5.8
Port Alfred	Plant	-	-	4.0	5.6	8.2
	Animal	-	-	0	11.3	8.2
Queenstown	Plant	-	-	-	6.8	7.0
	Animal	-	-	-	1.1	6.8
Mean	Plant	0	0	1.6	5.0	10.3
	Animal	1.2	0	0.4	5.4	4.8

## Discussion

South Africa is a highly biodiverse country with over 20,000 plant and 2,060 aquatic and terrestrial vertebrate species [30], which potentially provide an amazingly rich palette for inspiration, adoption and use within its many cultures, which can be manifest in names of landmarks, sites and urban streets. Indeed, the use of animal and plant names for street names was widespread both between and with towns, currently being at least 11.1% for plants and 5.3% for animals. It is not possible to adjudge if this is particularly high or low, because there is no comparative literature from other countries or regions. However, at the individual town level, there was high variation, with some towns (six) having no biodiversity street names, some being high for one but low for the other (e.g. Dordrecht had 42% of the streets named after plants, but none named after animals), and others being above the average for both (e.g. Bothaville had 22.5% of streets named after plants and a further 17.2% named after animals). Precisely why there is such high variation would require detailed archival research in each town. Almost one quarter of the towns had names reflecting the natural environment, mostly in terms of aquatic features such as springs or rivers, which may be a reflection of the importance of water sources in what is largely an arid to semi-arid country.

Contrary to the first hypothesis there was a higher prevalence of plant street names than animal ones. However, approximately half of the plant names were of non-native species, whereas only a small minority of animal street names were of non-native animals. The reasons underlying both these patterns are unclear. For the former it might be that plant names are intended to evoke a setting of beauty and a degree of serenity or harmony with the natural world to mimic the calming effects of nature on stress amongst urban residents [31,32]. For the latter, I hypothesise it is a reflection of the high diversity of large, charismatic mammals and avifauna native to South Africa. This could be tested through comparison to faunally depauperate regions. Amongst the plant names, most life forms and groups were well represented (other than vegetables), but this was not so for animal names, which were mostly of mammals and birds. Relatively few of the animal names were of smaller taxa and thus it is possible that steps could be taken to encourage the use of include smaller, less charismatic animal taxa, whether biocultural or economic keystone species or not [24], in street names.

The second hypothesis found some support, because there was a clear increase in the proportion of plant street names through time. Though the proportion of animal street names was a lot more variable through time, overall, there are more streets now that have animal names than was the case pre-1950. That one-fifth of the towns has more than 20% of their streets named after plants suggests that the trend of greater proportions of biodiversity names has potential to continue. This finding is potentially at odds with discourses relating to the extinction of experience in contact with and knowledge of biodiversity in urban settings. Thus, it will be necessary to dig deeper to understand how and why city authorities use particular biodiversity names.

The third hypothesis that the national biodiversity symbols would be prominently represented in the biodiversity street names was not strongly supported. The national animal (springbuck) was the tied third-most frequent animal street name, but no streets were named after the national fish and very few after the national bird. Protea was the second-most common plant street name, but not specifically the King Protea. The heritage status of the national symbols is also reinforced through their use in other spheres, such as the national cricket team being named the Proteas and the national rugby team are known as the Springbucks. Thus, the results suggest that more could be done to elevate the national biodiversity symbols in everyday visibility such as in street names.

Considering the more local scale, there was no association between the urban street names and the biome in which particular towns were located. In other words, there was no evidence that the people and institutions proposing street names were drawing from or inspired mostly by the local or unique species around them or in the vicinity of the town. Because of South Africa’s high diversity status (the only country with three global biodiversity hotspots) and diverse terrains and climates the levels of endemism are also high, but are not reflected in street names. Internationally, there is a growing move to define and legally protect the names of commercial products (especially drinks and foods) associated with particular place names (geographic indicators of origin). This allows for development of niche markets and branding [33], which also tie into rural development and promoting tourism to these regions [34]. It is conceivable that a similar concept (without legislation) could be promoted with regards to local biodiversity through towns having biodiversity street names that promote the fauna and flora particular to their area, which would be educational (for visitors and residents alike), build on local knowledge and heritage, but also feed into commercial opportunities and labelling. Indeed, local species are increasingly neglected with declining interactions with urban nature, and accessibility of video and visuals of internationally exotic and charismatic species [24]. Thus, local biocultural diversity is potentially at risk of being subsumed into a more global one.

In conclusion, street names offer an interesting reflection on what and who have been or are significant in the shaping of the national and local contexts of towns and cities. This mirror can be used to interpret changing attitudes through time and contestations of knowledge, heritage and values. With specific reference to biodiversity, street names are potentially one indicator of biocultural diversity as urban authorities and residents reflect on what species and forms are of interest or value to them. However, increased effort to name streets after species that cover a wider array of plant and animal groups, and that can be found locally is likely to improve a sense of identity and pride in local biodiversity and urban heritage. They provide an opportunity for presenting and conserving aspects of local culture related to biodiversity in settings where there are limited and decreasing interactions with nature.

## Supporting information

S1 Data Street names raw data. (XLSX)
